# Conflict-reducing innovations in development enable increased multicellular complexity

**DOI:** 10.1098/rspb.2023.2466

**Published:** 2024-01-10

**Authors:** Jack Howe, Charlie K. Cornwallis, Ashleigh S. Griffin

**Affiliations:** ^1^ Center for Evolutionary Hologenomics, Globe Institute, Copenhagen University, 1350 Copenhagen, Denmark; ^2^ Department of Biology, Lund University, 223 62 Lund, Sweden; ^3^ Department of Biology, University of Oxford, Oxford OX1 3SZ, UK

**Keywords:** germline, evolution, development

## Abstract

Obligately multicellular organisms, where cells can only reproduce as part of the group, have evolved multiple times across the tree of life. Obligate multicellularity has only evolved when clonal groups form by cell division, rather than by cells aggregating, as clonality prevents internal conflict. Yet obligately multicellular organisms still vary greatly in ‘multicellular complexity’ (the number of cells and cell types): some comprise a few cells and cell types, while others have billions of cells and thousands of types. Here, we test whether variation in multicellular complexity is explained by two conflict-suppressing mechanisms, namely a single-cell bottleneck at the start of development, and a strict separation of germline and somatic cells. Examining the life cycles of 129 lineages of plants, animals, fungi and algae, we show using phylogenetic comparative analyses that an early segregation of the germline stem-cell lineage is key to the evolution of more cell types, driven by a strong correlation in the Metazoa. By contrast, the presence of a strict single-cell bottleneck was not related to either the number of cells or the number of cell types, but was associated with early germline segregation. Our results suggest that segregating the germline earlier in development enabled greater evolutionary innovation, although whether this is a consequence of conflict reduction or other non-conflict effects, such as developmental flexibility, is unclear.

## 1. Introduction

Multicellular organisms vary greatly in multicellular complexity: some are relatively simple, containing fewer than a dozen cells with no discernible cell types, whereas others contain billions of cells and thousands of cell types [1]. The complete suppression of conflict among cells within an organism is predicted to be required for multicellular complexity to evolve [2–4]. However, the widespread variation in the size and complexity of multicellular organisms suggests that internal conflicts have been resolved to varying degrees in different lineages [5].

Conflict within a multicellular organism occurs when the inclusive fitness interests of the constituent cells are misaligned [6]—the cells are not all working towards the same goal. The mode of group formation demonstrates how such conflict can limit complexity. Obligate multicellularity, where cells can no longer survive and reproduce independently, has been key to the evolutionary diversification of numerous lineages across the tree of life, and has only evolved in groups that form by cell division [7]. Forming a multicellular group through cell division—where cells ‘stay together’—eliminates selection within a group by ensuring all cells are clonally related [8,9]. A gene that decreases the reproduction of an individual cell while increasing the total reproduction of the group will be favoured, as the interests of all cells are aligned in maximizing group fitness [9–11]. This enables altruistic traits like sterility to evolve, and ultimately complex adaptations such as eyes, wings or scales [4,12]. By contrast, multicellular groups that form by the aggregation of free-living cells may temporarily benefit from aggregation—through increased size [13] or genetic diversity [14,15]—but they cannot reliably eliminate conflict, as clonality is not guaranteed. As such, aggregative organisms have only ever evolved facultative multicellularity, and are much more limited in their complexity [7].

There remains, however, great variation in complexity among obligately multicellular groups that is not explained by the mode of group formation [7]. While significant variation in complexity will be due to direct selection on size and complexity, this could also suggest that variation in the levels of internal conflict could limit complexity. While clonal development creates groups without internal genetic variation, this is only ever temporary. Errors in DNA replication inevitably occur during cell division, which generates the material for within-group selection that can produce selfish-cell lineages to the detriment of the group. Cancer is perhaps the most salient example of this: mutations enable rapid cell division that is favoured by within-organism selection, despite their harm to the organism [16].

Multicellular organisms have therefore evolved conflict resolution mechanisms that align inclusive fitness interests by shifting selection from acting within, to acting among, multicellular groups. Variation in such mechanisms could generate variation in the levels of internal conflict and therefore the potential complexity that an organism can achieve. Two common mechanisms of conflict suppression in multicellular organisms are single-cell bottlenecks [2,7,17] and a strict separation of germline and somatic cell lineages [18–20]. A single-cell bottleneck suppresses conflict by resetting clonality and segregating mutations among offspring [21,22]. Were a selfish mutant to arise within an organism, it could spread by parasitizing the cooperation of other cells in order to become overrepresented in the reproductive propagules. In the next generation, however, the multicellular group would contain only mutant selfish cells. With no cooperative cells to parasitize, such groups would perform poorly relative to groups composed only of cooperators [21,23,24]. Consequently, selfish mutants are exposed to selection and will be selected against at the organism level. Conversely, a beneficial mutation will also be exposed to selection and can more easily spread—it too will be present in all cells in the following generation [21,22]. Development from a single cell may also provide greater developmental flexibility and potential for evolutionary innovation than one limited by a complex bodyplan already in place [24,25].

A strict separation between germline and soma can also suppress conflict by limiting the evolutionary potential of mutations [19,20,26,27]. Many multicellular organisms demarcate a small subset of cells early in development as the germline that will eventually produce the gametes, which we refer to as early germline segregation. *Caenorhabditis elegans*, for example, sets aside its germline after only four cell divisions [28]. If these cells are removed, the nematode develops normally but lacks gametes [28]. By segregating the ‘mortal’ soma from the ‘immortal’ germline, any mutations that arise within the growing soma are removed as the soma perishes with each organismal generation. The window for mutants to arise and access the germline is therefore limited by the duration before germline segregation: the earlier germline segregation occurs, the shorter the potential period of conflict. The alternative, germ cell differentiation late in development, allows somatic mutations to enter the germline and could create potential conflict over producing germline versus soma. A selfish mutant might preferentially contribute to the immortal germline rather than the mortal soma [19,29], as observed in the selfish lineages of colonial ascidians that produce only germ cells without contributing to the soma after fusing with other colonies [30,31] . By limiting the window for mutations to occur in the germline, early germline segregation acts to limit the per generation mutation rate—later somatic mutations cannot enter the germline, and the inactive germline progenitor stem cells are protected from replication errors. Species that lack early germline segregation and instead differentiate germline cells throughout adulthood, such as plants, have a correspondingly higher per generation mutation rate [32], although some groups appear to have mechanisms that prevent this [33].

Humans use both mechanisms during development: we start from a single zygotic cell and mark a small subset of cells as the germline stem cells long before reproductive maturity. Many model organisms do similarly. But this is not representative of multicellular organisms, nor is it particularly representative of the Metazoa [34]. Many organisms do not have a single-celled bottleneck that separates each generation, or early segregation of the germline and soma. Instead, they often reproduce with multi-celled propagules (through budding or fission) and have totipotent stem cells that contribute to both the germline and soma as adults. *Hydra*, for example, often reproduces by budding—forming a miniature individual that is released fully-formed—and has widely distributed adult stem cells that contribute to both the germline and the soma [35]. Therefore, in *Hydra*, reproductive propagules can be genetically heterogeneous, increasing the potential for conflict, although occasional sexual reproduction re-establishes clonality [36]. Similar examples can be found in many animal lineages, from the early-branching phyla like the Cnidaria and the Porifera, to our closer relatives in the Chordata [30,34], as well as in many plants, fungi and algae [37]. Strict, single-cell bottlenecks every generation and the early segregation of germlines, however, have evolved independently multiple times—notably in the Metazoa with the Craniata, Ecdysozoa and Mollusca [34]. All multicellular organisms, therefore, can be considered to fall on a continuum of potential conflict depending on the relative frequency of single-celled bottlenecks and at what stage the germline is specified [18].

Despite the proposed importance of regular bottlenecks and early germline segregation for explaining variation in multicellular complexity, empirical tests are lacking. Here, we use a phylogenetic comparative approach to test whether multicellular complexity is explained by the presence of a strict single-celled bottleneck, and the presence and timing of a germ–soma divide. To quantify multicellular complexity, we collated data from the literature on the number of cells and the number of cell types. We combined this with life cycle accounts to determine the presence or absence of a strict single-cell bottleneck and the timing of germline segregation across all major obligately multicellular lineages. In total, data were obtained for 138 species spanning the Bacteria, Chromista, Plantae, Fungi and Animalia.

## 2. Methods

### (a) Data collection

Data previously collected by Fisher *et al.* formed the basis for the current analysis [7]. They included published data on cell numbers and the number of cell types (e.g. [5,38,39]). While Fisher *et al.* [7] considered obligately and facultatively multicellular organisms, we only considered obligately multicellular organisms. We expanded this dataset to include information on the ability of organisms to reproduce without single-cell bottlenecks, and the presence and timing of germline sequestration. For each entry in the original dataset, we conducted a search on Web of Science for terms related to different modes of reproduction: ‘(ALL = (reproduct* OR sex* OR asex* OR vegetat* OR fissi* OR clonal* OR regenerat* OR rhizo* OR germ-line* OR germline* OR germ line* OR bud* OR fragment* OR parthenogen* OR stolon*)) AND (ALL = TAXON)’. We selected relevant literature by screening titles and then the abstract. Additional literature was located by examining citations within relevant papers and using reviews, specifically [40] for animals, [39,41,42] for algae and [38] for cyanobacteria.

We defined organisms as having a strict single-cell bottleneck between generations when there were no examples of fissiparous or budding reproduction in the literature, including when reproduction was triggered by fragmentation of the organism. This means that those organisms which have not been subject to in-depth study, or that rarely reproduce by fragmentation, may be more likely to be erroneously categorized, which could reduce our ability to determine the evolutionary consequences of fissiparous reproduction. Similarly, some species contain populations or subspecies that are exclusively sexual while others are exclusively asexual, although the planarian flatworm *Schmidtea mediterranea* was the only example in our dataset [43]. In this case, we denoted the organism as not having a strict bottleneck and repeated our analyses with this datapoint reversed. For some groups, such as red algae, there are sexual, asexual and budding/fissiparous life cycles that exist in parallel [42,44]. We classified these as lacking a strict bottleneck given that the fissiparous life cycle can continue indefinitely. By contrast, if the sexual and fissiparous stages strictly alternate as part of the life cycle (e.g. in parasites such as schistosomes [45]), we considered this as including a strict bottleneck, although there were no examples of such life cycles in our dataset.

For the timing of germline segregation, we considered two possible conditions: (i) Early germline segregation. We considered germline segregation to be ‘early’ if it is segregated before reproduction, and stem cells present in the adult organism contribute to *either* the soma *or* the germline, but not both. In well-documented cases, early germline segregation is demonstrated by removing germline progenitors resulting in an inability to regenerate, as in mice or *C. elegans* [28]. However, such experiments have been conducted in only a few model organisms. (ii) Late germline differentiation. Organisms were considered to have ‘late’ germline differentiation if adult stem cells contribute to both the germline and the soma. This is often demonstrated as the regeneration of the germline if it is removed in adults, or the generation of a new germline in a fragment of an individual that did not contain the gametes. As we are only interested in the timing of germline specification, we excluded organisms that do not have a soma–germline separation, and were agnostic to the mechanism that determines the germline stem-cell progenitors [46]. Germline data for the Volvocine algae were from [39], and for metazoans were from [34,46,47]. All references are available with the data uploaded at github (https://github.com/jackhowe-bio/complexity_project).

We collected all data at a species level where possible, but if this was lacking we used information for congeners. If there were no data available for the genus, the species was removed from that analysis. Measured traits were generally very similar for congeners, although exceptions do occur, for example, flatworms in the genus *Schmidtea* have both sexual and fissiparous reproduction [43,48]. We therefore ran sensitivity analyses checking the consistency of results when taxa with inferred data from congeners were removed (see Statistical analyses (§2c)).

### (b) Phylogeny

A phylogeny of the species in our dataset was generated using the ‘R Tree of Life Project’ (rtol) [49]. Tree branch lengths were generated as described by Grafen & Hamilton [50] and branches shorter than 10^−25^ were removed, creating multichotomies. Multichotomies in the tree were randomly resolved.

### (c) Statistical analyses

Bayesian phylogenetic mixed models (BPMMs) implemented in MCMCglmm [51] in R 4.2.0 [52] were used to conduct five sets of analyses to estimate the following. (i) The effect of a strict single-celled bottleneck separating each generation (two-level fixed factor) on cell number (Poisson error distribution). (ii) The effect of a strict single-celled bottleneck separating each generation (two-level fixed factor) and the number of cells (log-transformed continuous fixed effect) on the number of cell types (Poisson error distribution). The number of cells was included as a fixed effect as it is known to correlate with the number of cell types [7]. (iii) The effect of germline segregation timing (two-level fixed factor) on cell numbers. (iv) The effect of germline segregation timing (two-level fixed factor) and cell number (log-transformed continuous fixed effect) on the number of cell types. (v) The phylogenetic correlation between the presence of a strict single-celled bottleneck (binomial error distribution) and early germline sequestration (binomial error distribution) using multi-response BPMMs. In all models, the evolutionary relationships between organisms were modelled by fitting a phylogenetic variance–covariance matrix, constructed from the phylogeny as a random effect. The phylogenetic signal in Gaussian and Poisson response variables was calculated as the phylogenetic variance divided by the total random effect variance (‘Phylogenetic heritability’ in the terminology of MCMCglmm [51]). For binary traits, the residual variance is not identifiable and was fixed to 1. Phylogenetic signal for binary response variables was therefore calculated using the intraclass correlation coefficient defined as: phylogenetic variance/(phylogenetic variance + 1) + *π*^2/3^ [51,53].

For models with Poisson error distributions, weakly informative inverse-gamma priors were used for the random phylogenetic effects (*V* = diag(*n*), *ν* = *n* − 1 + 0.002, where *n* is equivalent to the number of response traits). For models with binary error distributions, parameter-expanded priors were used (*V* = diag(*n*), *ν* = *n* − 1, *αμ* = rep(0, *n*), *αV* = 1000) that have a lower pull toward zero. For fixed effects, non-informative uniform priors were used (MCMCglmm defaults). We tested the sensitivity of our results to prior specification by reconducting all analyses varying the value of the shape parameter (*ν* *=* 1 and *ν =* 2)*.* (See the electronic supplementary material for descriptions of all models and priors.) In addition, we tested the sensitivity of our results to inferring data from congeners by re-running analyses with the datapoints inferred from congeners removed. We also tested whether our results were specific to the Metazoa by re-running analyses without the Metazoa, and only using data on Metazoa. All models, and their results, are reported in the electronic supplementary material, information.

We optimized model settings by running the first analysis with varying number of iterations (5 × 10^5^–10^7^), burn-in lengths (10^4^–10^6^), and thinning factors (100 and 1000), and chose the combination of parameters that minimized the autocorrelation of successive sampled means and variances (8 × 10^6^ iterations, 10^6^ burn-in iterations and thinning factor of 1000 for all models apart from 1.6 × 10^7^ iterations when testing for a phylogenetic correlation between a strict bottleneck and early germline segregation). Visual inspection of traces and the Gelman–Rubin convergence diagnostic [54] confirmed that chains converged for all models (potential scale reduction factor less than 1.05 in all cases). Differences between parameters (e.g. the presence and absence of single-celled bottlenecks; correlations tested against 0) were deemed significant when 95% credible intervals (CI) did not overlap with 0 and the reported pMCMC value was less than 0.05.

Ancestral states were estimated for germline timing (binomial error distribution) and the presence of strict single-celled bottlenecks (binomial error distribution) using intercept-only BPMMs. Ancestral states for the number of cell types adjusted for number of cells were estimated using a BPMM of cell-type number with number of cells fitted as a fixed effect. Traits and reconstructions were plotted across the phylogeny using ggtree [55], and figures were produced using ggplot2 [56]. All code and data are available at Github (https://github.com/jackhowe-bio/complexity_project) as well as in Dryad [57].

## 3. Results

The absence of a strict single-cell bottleneck separating each generation is common throughout multicellular organisms. It predominates in the Streptophyta (plants), the Rhodophyta (red algae) and the Phaeophyta (brown algae), and is common in the early-branching lineages of Metazoa. The Chlorophytes, sister species to the Streptophytes within the Viridiplantae, represent an exception in our dataset, where there all species included use a strict bottleneck. While it is possible that Chlorophytes differ substantially in their biology compared with other green algae, this pattern likely arises from an overrepresentation of Volvocales species in the literature, as they are a model system for studying the evolution of multicellularity. In our dataset, ancestral state reconstructions indicated that there was a lack of a strict generational bottleneck at the root of the tree. However, as the major branches in the dataset evolved multicellularity independently, it is more relevant that the Metazoa, Fungi and Plantae were all predicted to have evolved from an ancestor without a strict single-celled bottleneck. Conversely, the Volvocales, Rhodophyta and Ocrophyta were predicted to have evolved from an ancestor with a strict bottleneck. We find multiple transitions between single-cell bottlenecks and fissiparous reproduction, but the exact number of predicted gains and losses are heavily dependent on the phylogenetic sampling of organisms, and the sparse and biased data available, so the number and timing of transitions here are unreliable.

**Figure 1.**
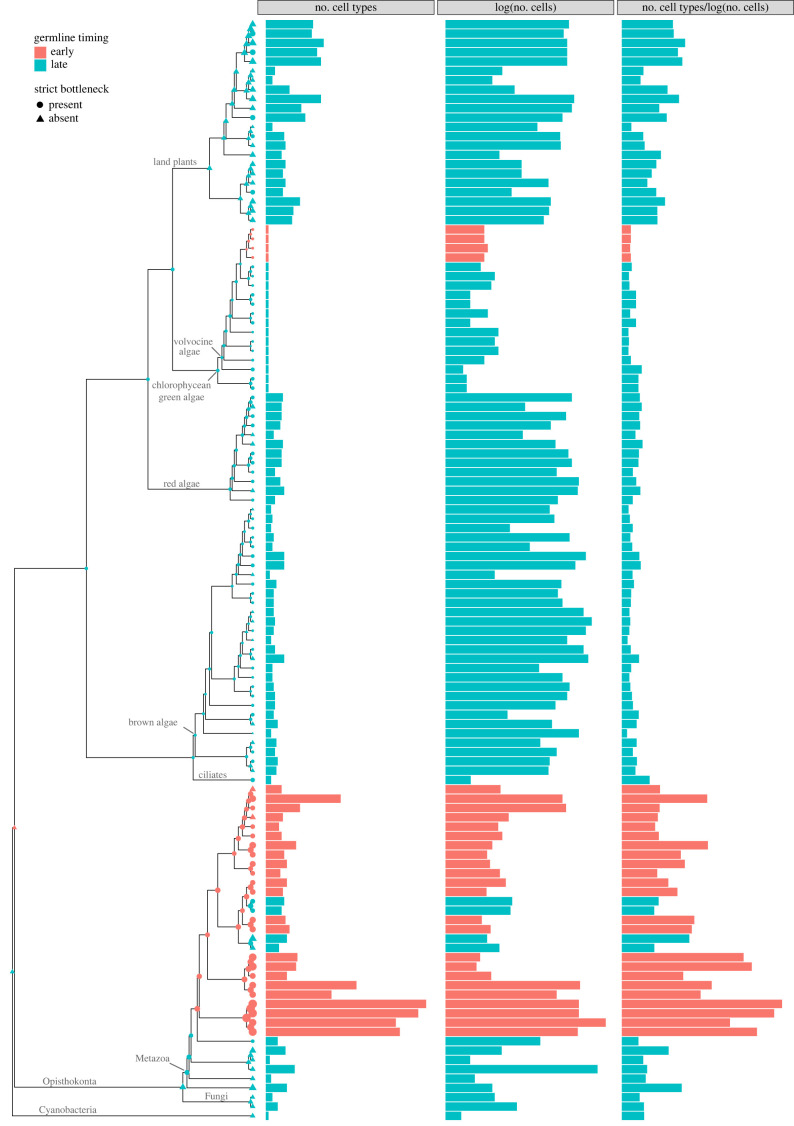
The phylogenetic distribution of the presence of strict single-cell bottlenecks, early germline segregation, the number of cells, the number of cell types and the number of cell types after correcting for the number of cells. Points on the phylogeny show ancestral state reconstructions (red, early germline; blue, late germline; triangles, no bottleneck; circles, bottleneck) with size proportional to number of cell types after controlling for cell number.

The timing of germline specification was less evolutionarily labile than the presence of single-celled bottlenecks. Late germline differentiation was most typical, and is observed in all Plantae, Rhodophyta, Ocrophyta and Fungi and in the early-branching Metazoa. Early germline segregation during development was observed in only five groups represented in our dataset: one in the volvocine algae, and four in the Metazoa (Craniata, Ecdysozoa, Mollusca and Annelida). Ancestral state reconstruction estimated this to be a single gain of early germline segregation in the Metazoa, with two subsequent losses and a single gain of early germline segregation in the volvocine algae (figure 1).

**Figure 2.**
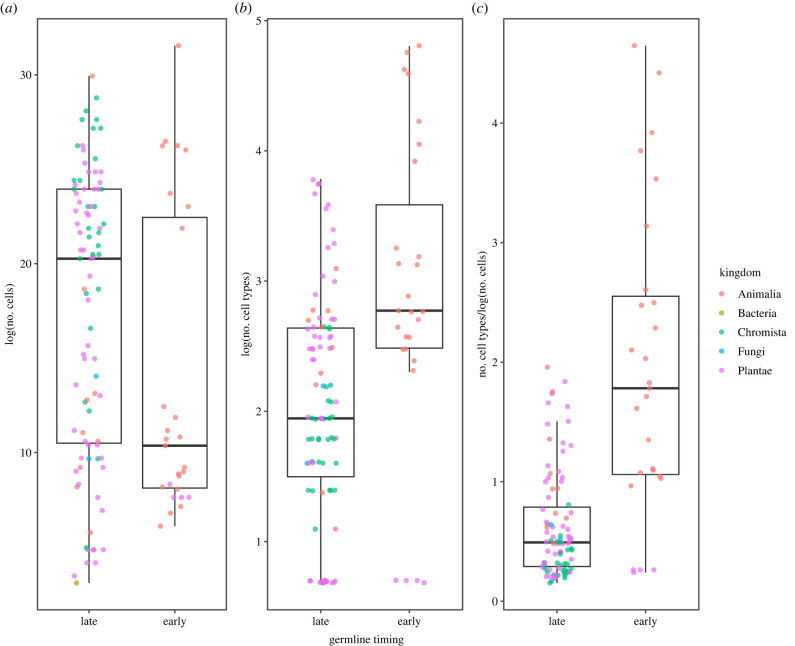
Association between the timing of germline segregation and measures of multicellular complexity. Cell number (*a*) tends to be greater in organisms with a late-segregating germline compared with organisms with early-segregating germline, although not significantly so after accounting for phylogeny (95% CI = −4.81 to 4.3, pMCMC = 0.929). In contrast, organisms with an early-segregating germline have a significantly greater number of cell types (*b*) and cell types after controlling for cell number (*c*) (95% CI = −0.84 to −0.11, pMCMC = 0.012). Colours indicate kingdoms included in the analysis.

The evolution of an early-segregating germline was significantly correlated with an increase in the number of cell types, after controlling for the number of cells (figures 1 and 2) (early versus late germline, 95% CI = −0.84 to −0.11, pMCMC = 0.012, electronic supplementary material, table S110). Given our analyses included cell number, we further investigated whether early germline segregation influenced the number of cell types *per se* or the relative number of cell types for a given cell number. While organisms with late germline segregation have generally more cells, the timing of germline segregation was not significantly related to cell number in our phylogenetically controlled analysis (figure 2*a*, 95% CI = −4.81 to 4.45, pMCMC = 0.933, electronic supplementary material, table S14). As a result, the number of cell types remained positively associated with early germline segregation when cell number was removed from the analysis, although the relationship was much weaker (pMCMC = 0.075–0.127 depending on the priors, electronic supplementary material, tables S115–S120).

The association between germline timing and number of cell types after controlling for cell number was largely driven by Metazoa: the relationship disappeared when data from Metazoa were excluded (95% CI = −0.66 to 1.1, pMCMC = 0.669, electronic supplementary material, table S170), but remained significant when only data from Metazoa were included (95% CI = −1.03 to −0.13, pMCMC = 0.018, electronic supplementary material, table S140). Furthermore, similar to the overall dataset there was no significant association between early germline segregation and number of cells in the Metazoa (95% CI = −8.74 to 8.8, pMCMC = 0.926, electronic supplementary material, table S134).

**Figure 3.**
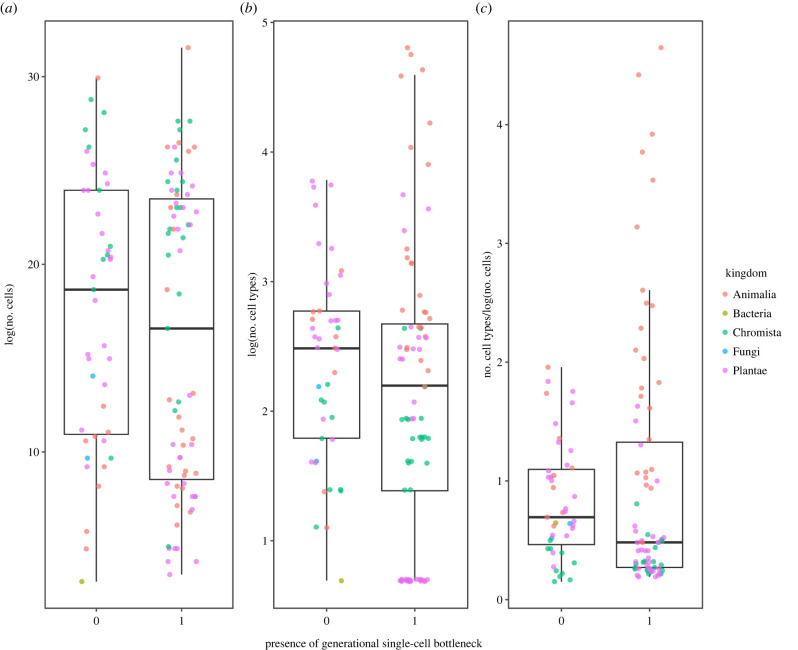
Association between a strict single-cell bottleneck separating organismal generations and measures of multicellular complexity. There was no association between the presence of a strict single-cell bottleneck and (*a*) number of cells (presence versus absence, 95% CI = −0.83 to 3.68, pMCMC = 0.197), (*b*) number of cell types (not tested) or (*c*) number of cell types after controlling for cell number (95% CI = −0.13 to 0.23, pMCMC = 0.573). Colours indicate kingdoms included in the analysis.

**Figure 4.**
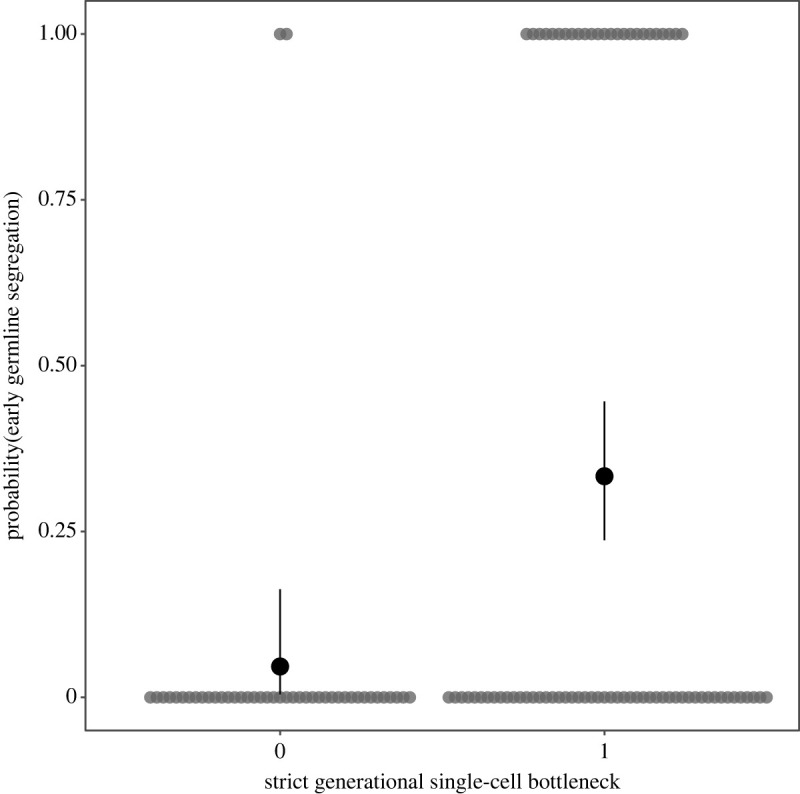
The relationship between single-cell bottlenecks and early germline segregation. There is a significant positive association between a strict single-celled bottleneck separating each organismal generation and the early segregation of the germline (posterior mode for correlation coefficient = 0.74, 95% CI = 0.37–0.98). Grey dots represent species (jittered to reduce overlap) and black dots with error bars show means and 95% binomial confidence intervals calculated using the Agresti–Coull method.

There was no significant relationship between the presence of a strict single-cell bottleneck between generations and either the number of cells (95% CI = −0.83 to 3.68, pMCMC = 0.197, electronic supplementary material, table S92) or cell types (figure 3, 95% CI = −0.13 to 0.23, pMCMC = 0.573, electronic supplementary material, tables S91 to S102). Strict single-celled bottlenecks were nevertheless positively phylogenetically correlated with early germline segregation (figure 4; electronic supplementary material, data). This suggests that single-celled bottlenecks might not influence multicellularity complexity directly, but rather could be important in generating an early germline, which in turn suppresses conflict enough to allow differentiated cell types evolve.

All results remained consistent across different priors and when data for species with missing data were inferred from congeners (electronic supplementary material, tables S1–S30).

## 4. Discussion

The evolution of obligate multicellularity depends on the low-conflict conditions that clonal development creates [2–4]. Mutation, however, inevitably erodes clonality and provides the material for within-organism selection to favour non-cooperative cells. Low-conflict conditions are re-established by regular single-cell bottlenecks and a strict segregation of the germ and soma, shifting selection to among multicellular groups, rather than among their cells. Yet many multicellular organisms use neither, and might therefore be expected to suffer from internal conflict [18]. Using a dataset spanning multiple origins of obligate multicellularity [7], we tested whether the evolution of strict single-celled bottlenecks and strict germlines facilitated greater cooperation and thereby multicellular complexity—measured as the number of cells and cell types. Organisms with early-segregating germlines contained more cell types, but not more cells, than organisms with a late-segregating germline or organisms lacking a clear germline (figures 1 and 2). Organisms with strict bottlenecks contained no more cells or cell types than organisms that reproduce through fission (figure 3). Early germline segregation and the use of a strict single-celled bottleneck each generation were also significantly correlated (figure 4). These results suggest that developmental innovation may be as important as conflict limitation in the evolution of multicellular complexity.

Our ability to conduct phylogenetic analyses of patterns in development is currently limited by the amount and quality of available data. This is partly the result of the challenges involved in measuring relevant variables. Estimates of cell number and cell-type number used here were based on morphological observations by multiple authors [5,7]. While such estimates are often dependent on several assumptions, they capture the broad patterns of size and complexity across multicellular life and are comparable across species, providing valuable insights into evolutionary patterns [7,58].

Organisms with and without strict bottlenecks every generation have similar cell numbers and cell types (figure 4). It appears, therefore, that the potential internal conflict in organisms capable of fission is sufficiently suppressed to have not limited the evolution of multicellular complexity. An occasional bottleneck may be sufficient to suppress conflict in taxa that are mainly fissiparous, and therefore need not occur every generation in order to maintain cellular cooperation. Indeed, simple models have suggested that only infrequent bottlenecks are required to prevent cell selfish lineages from persisting in a population [59]. Few data exist on the relative frequency of bottlenecks, however, so this cannot currently be tested. Groups that are obligately fissiparous may provide some insight, but these are rare, typically derived states that have not produced large radiations, and often considered to be evolutionary dead-ends owing to their lack of sexual recombination [60]. A single-cell bottleneck often occurs in tandem with sexual reproduction, although single-cell propagules can be asexually produced spores or similar. The benefits of sexual reproduction [61]—for example, recombination enabling the accumulation of beneficial and removal of deleterious mutations [62], while preventing the spread of selfish genetic elements [63]—are distinct from the benefits we discuss here, but may provide an additional selection pressure that favours a bottleneck where sex results in the formation of a single zygotic cell.

Bottlenecks could also occur through means not captured here. Cryptic bottlenecks, where a single cell initiates a multicellular propagule prior to release from the mother group, also suppress conflict. For example, in many volvocine algae, a single cell divides to form a daughter colony while still physically within the parental alga, before being released in a process analogous to live-bearing animals [64,65]. In these cases, the next generation still starts from a single cell, but this is not clear in many other organisms, such as plants that spread vegetatively. Structural organization can also create bottlenecks. Many organisms with non-deterministic growth, such as plants and fungi, are modular in that they comprise semi-independent, repeating parts. The branches of a plant start from a single, or very small group of meristematic stem cells [66,67], while branching fungal hyphae spread radially. Both growth patterns would segregate genetic variation, shifting selection to act among modules rather than within them (e.g. [33]).

Fusion among modular organisms—observed in red algae [68], fungi [69], sponges [70] and colonial ascidians [29,30]—bypasses the single-cell bottleneck and provides another source of genetic variation. This variation enables the evolution of parasitic genotypes, which have been observed in colonial ascidians [31] and fungi [69,71]. Fusion is therefore limited to between close relatives by polymorphic allorecognition loci, and it is not clear how often between-, rather than within-, clone fusion occurs outside of the laboratory [69]. The data are not currently available to test whether the conflict created by fusion limits complexity in these organisms, as in aggregative multicellularity [7].

Early germline segregation prevents mutations from persisting beyond a single generation by excluding any somatic mutations from the germline once it has been segregated, thereby removing the evolutionary benefits for potential somatic cheats [20]. The observation that organisms with an early-segregating germline generally have more cell types—driven by the correlation in Metazoa—is consistent with conflict suppression enabling the evolution of greater cooperation. A potential alternative non-conflict-reducing benefit to early germline segregation would be if maintaining a cell's pluripotency limits its developmental potential, which early segregation may guard against. Consistent with this idea, the mechanism of germline specification has previously been linked to species radiation rates in the Amphibia: maternal specification (preformation) of the germline is associated with higher speciation rates than those that rely on cell–cell communication (induction) [72] (although see [73]). This effect is not seen, however, in molecular rates of evolution of developmental gene networks [74]. While preformation of the germline tends to occur earlier than induction, both still occur early in development, so the effect of timing of germline specification is untested. Under a conflict scenario, one might expect groups with late germline differentiation to suffer from a greater mutational load, as somatic mutations enter the germline (e.g. [32]), whereas in a release of constraints scenario, there may be a slower rate of evolution in early developmental genes.

Clearly, something special has occurred during the evolution of the Metazoa. They have reached a maximum of many more cell types for their size than observed in other groups, and they are the only group that shows a significant correlation between germline timing and the number of cell types. The only other transition to early germline specification is observed in the volvocine algae, and a concomitant increase in complexity has not followed. Without other groups exhibiting transitions to early germline specification, we cannot rule out the possibility that another, uniquely animal trait besides early germline specification confounds our analysis, but this does not preclude the potential importance of early germline specification in the increase in complexity in the Metazoa. While there are insufficient transitions in germline states to conduct a formal analysis, ancestral state reconstructions support that early germline specification facilitated the evolution of greater levels of cellular complexity: early germline specification preceded the increases in cellular complexity in each case. The common ancestor of the Metazoa was also estimated to have had late germline differentiation and the ability to reproduce through fission—in agreement with previous analyses [34]—and we identified only a single transition to early germline specification in the animals, with three subsequent losses. This is likely an artefact of the animal species for which we have complexity data as other studies with wider metazoan sampling suggest early germline specification has evolved independently in different groups [46,75].

The effect of germline specification timing and strict single-cell bottlenecks on the number of cell types could also be explained by the limitations of modular versus unitary, deterministic body plans. Germline segregation in adult organisms through broadly distributed pluripotent cells is common in modular organisms, necessarily so: there must be a totipotent stem cell in each module that produces the somatic cells and eventually the germline. The wide distribution of totipotent cells enables reproduction through budding and fission, as all missing tissues and cell types can be replaced. The positive correlation between the presence of a strict single-cell bottleneck between generations and early germline specification supports this (figure 4). Such modular organisms can reach great sizes by the repetition of similar subunits, which increases cell number without a concomitant increase in the number of cell types. Indeed, organisms with a late-segregating germline and those capable of bypassing a single-celled bottleneck tend to comprise more cells than those with an early-segregating germline and a strict single-celled bottleneck, albeit non-significantly so once phylogeny is accounted for. Within the Metazoa, there was no association between germline timing and cell number, suggesting that the observed increase in cell-type number is driven predominantly by greater cell-type diversification in animal lineages with early germline specification, rather than a decrease in the number of cells (figures 2 and 3).

## 5. Conclusion

We found no evidence that the evolution of an obligate single-cell bottleneck separating each generation has enabled greater complexity. Organisms where each generation is separated by a single-cell bottleneck, do not contain more cells or cell types, but do have germlines that segregated earlier in development. However, in a pattern driven by the Metazoa, we observed that organisms with an early-segregating germline possessed a greater diversity of cell types, albeit potentially fewer cells. An increase in cell types associated with early germline segregation suggests that it may have been an important innovation in the evolution of the Metazoa, but whether this complexity is driven by conflict suppression, greater developmental flexibility or both is difficult to separate.

## Data Availability

All code and data are available at Github (https://github.com/jackhowe-bio/complexity_project) as well as from the Dryad Digital Repository: https://doi.org/10.5061/dryad.7wm37pvzf [57]. Supplementary material is available online [76].
